# Persistent catatonia following epileptic seizures: a case report and systematic literature search

**DOI:** 10.1186/s12888-018-1935-0

**Published:** 2018-10-29

**Authors:** Ragnar Verbraeken, Jurjen J. Luykx

**Affiliations:** 1 OPZ Geel, Geel, Belgium; 2 Department Of Psychiatry, Rudolf Magnus Institute of Neuroscience, Utrecht University, Utrecht, The Netherlands; 3 Department of Translational Neuroscience, Rudolf Magnus Institute of Neuroscience, Utrecht University, Utrecht, The Netherlands; 4 SymforaMeander Hospital, Amersfoort, The Netherlands; 5 Department of Psychiatry, ZNA Hospitals, Antwerp, Belgium

**Keywords:** Postictum, Delirium, Catatonia, Lorazepam

## Abstract

**Background:**

Catatonia is frequently associated with mood and psychotic disorders as well as with general medical conditions, especially with seizures. In the case of the latter, catatonia mostly resolves when the seizures respond to the anticonvulsive treatment. We report, to our knowledge, the first case of a patient without affective or psychotic disorder, who developed catatonia in the postictum and whose catatonia did not resolve with anticonvulsive treatment, but did so with lorazepam.

**Case presentation:**

We describe a 36-year-old man, with no psychiatric history, except for a possible disorder in the use of cannabis, who developed catatonia after epileptic seizures. The catatonia did not respond to the anticonvulsant therapy, but did so to lorazepam 17 mg/d. Lorazepam could be tapered slowly and stopped without reemergence of catatonic signs.

**Conclusion:**

Catatonia should be part of the differential diagnosis in patients with bradyphrenia and/or remarkable postictal behavior. This report shows that lorazepam should be taken into consideration (before moving to ECT), in cases of unresolved catatonia, even if the seizures are reduced with anticonvulsants.

## Summary

We describe the first case of persistent catatonia following an epileptic seizure (ES) of a patient without a history of psychotic or affective disorders. The catatonic symptoms did not go into remission until a dose of lorazepam 17 mg daily was reached. We tapered and finally discontinued lorazepam without the patient developing new signs of catatonia.

## Background

Catatonia is a psychomotor symptom cluster first described by C. Wernicke (motility psychosis) and by K.L. Kahlbaum in 1874 in his publication ‘Die Katatonie oder das Spannungsirresein’ in which he regarded ‘being extremely tense’ as the main characteristic of catatonia [1]. Catatonia is observed in a plethora of psychiatric disorders, primarily mood and psychotic disorders, but sometimes in cases of, for example, autism [2–5] or obsessive compulsive disorder (OCD) [6] as well. In DSM-5 it has become possible to classify catatonia as ‘catatonia as specifier’ or ‘catatonic disorder not otherwise specified’ or ‘unspecified catatonia’. Furthermore, catatonia also occurs in non-psychiatric disorders such as neurological disorders, in metabolic diseases and intoxications, as a consequence of medication [5, 7–9], postoperatively [5], or after withdrawal of alcohol [10], benzodiazepines [8] or cannabis [11]. To our knowledge, catatonia following an ES had not been described before the current report. Here, by describing a case and a systematic literature search we raise awareness of this possible postictal condition to enable earlier diagnosis and management.

## Case presentation

A 38- year-old man, with no psychiatric history, known to suffer from epilepsy but who stopped his antiepileptic drugs (AED) is brought into a community hospital’s emergency room by an ambulance after being found in the streets in a confused condition and with reduced awareness. The history taking in the emergency room is hampered by bradyphrenia and amnesia. The patient merely relates a vague account of a pub fight. At that instant he does not know where he lives nor is he able to provide any contact information about relatives or friends. General physical examination, neurological examination, urine toxicology, brain CAT and cerebrospinal fluid findings (proteins and cells, mainly to exclude meningitis/encephalitis) are unremarkable. Blood analysis shows slight signs of leukocytosis. The EEG shows general slowing without traces of epilepsy. The patient is admitted to the neurology department for diagnostic work-up.

The next day the patient is somewhat less slow and more aware, although still unaware of the events on the day prior to admission. He believes to have fallen as a consequence of an ES. He indicates he has not been taking his AED for some time. Valproic acid was therefore restarted at a dose of 1500mgs daily. He denies having used any drugs or alcohol, as confirmed by urine toxicology. A new blood analysis is carried out, showing a decrease in leukocytosis, an increase of CK to 521 U/L and a slight increase in CRP to 16.2 mg/L. Brain MRI is unremarkable.

Two days after his admission an ES is observed by nursing staff and is interpreted as tonic-clonic, generalized. No clear underlying cause is identified. Valproic acid blood levels are 54 mg/L (at the time of admission this was < 10 mg/L), leading us to increase the dose to 2x1000mg/d. The EEG is unchanged.

The patient then shows peculiar behaviour: he stares and speaks little, leading the neurologist to suspect a psychotic episode and therefore a psychiatrist is consulted. During the psychiatric history the patient informs him that he does not have any complaints other than noticing being somewhat slow. He himself attributes this to the restarted AED. During psychiatric examination we notice bradyphrenia. The patient does blink his eyes and moves slowly, though fluently. There is no posturing or grimacing. He is friendly and cooperative. His conscienceness is clear and his attention span is undisturbed. He is well-oriented. Perception, memory and mood appear intact. The affect is flattened. He denies having suicidal thoughts.

Given this symptomatology the differential diagnosis at that stage included side effects of the valproic acid and, although there were insufficient DSM-5 criteria for catatonia at the time, catatonia. The attending psychiatrist decided to re-evaluate the patient a few days later.

Upon that re-evaluation, the patient rubs his hands in a stereotypical manner and there is evidence of grimacing and posturing (the patient sits bent over for minutes, with hands, head and shoulders in the same position) and of ambitendency. There is no Gegenhalten, no negativism, no Mitmachen. The Bush Francis catatonia rating scale (BFCRS) scores: 0 1 0 1 2 1 0 2 0 0 0 0 0 0 2 0 0 0 3 0 0 0 0 = 12 (Table 1). Again, the EEG remains without abnormalities and unchanged. Given the stereotypies, the bradyphrenia, hypoactivity, inappropriate behaviour, ambitendency and posturing combined with a BFCRS score of 12, we concluded the patient was suffering from postictal catatonia becoming more severe within 8 days following a seizure. To confirm this hypothesis, we treated him with lorazepam 3dd1mg and 1dd2.5 mg, without improvement, but the lorazepam didn’t bring about any sedation. He was then transferred to the psychiatry department for further diagnostic work-up and treatment. During the first days of his stay at the ward symptoms and signs were unchanged. A fourth EEG showed no change either. Lorazepam was increased by 3 mg every 2 days until at a total dose of 17 mg/d the patient clearly improved: he became more talkative, started moving more fluently and has not shown signs of grimacing or posturing since. His mood has remained cheerful. Then, lorazepam was gradually tapered by 1 mg/d until it could be stopped completely, with the patient remaining stable and free of symptoms. On his day of discharge at 0 mg of lorazepam an EEG showed a normal base rhythm with only a couple of short episodes of general slowing.

**Table 1:** Overview of the catatonic features during the course of illness. Day numbers refer to the number of days after the epileptic seizure

	Day 8 (0 mg Lorazepam)	Day 12 (11.5 mg Lorazepam)	Day 16 (17 mg Lorazepam)	Day33 (0 mg Lorazepam
Excitement	0	0	0	0
Immobility/stupor	1	1	0	0
Mutism	0	0	0	0
Staring	1	1	0	0
Posturing/catalepsy	2	1	0	0
Grimacing	1	1	0	0
Echopraxie/echolalia	0	0	0	0
Strereotypy	2	1	0	0
Mannerisms	0	0	0	0
Verbigeration	0	0	0	0
Rigidity	0	0	0	0
Negativism	0	0	0	0
Waxy flexibility	0	0	0	0
Withdrawal	0	0	0	0
Impulsivity	2	1	0	0
Autonomic obedience	0	0	0	0
Mitgehen	0	0	0	0
Gegenhalten	0	0	0	0
Ambitendency	3	0	0	0
Grasp reflex	0	0	0	0
Perseveration	0	0	0	0
Combativeness	0	0	0	0
Autonomic abnormality	0	0	0	0
Total	12	6	0	0

## Discussion

Over forty different catatonic symptoms have been described to date [2, 7, 12]. These may be subdivided into the following four categories: motor phenomena (stupor, catalepsy, rigidity, flexibilitas cerea), withdrawal (mutism, staring gaze, negativism, refusal to eat and drink), excitement (hyperactivity, combativeness, autonomous instability, undirected potentially dangerous aggression) and odd repetitive behaviour (grimacing echolalia, echopraxia, stereotypies, manierisms, perseveration, command-automatism, Mitmachen and Gegenhalten, and ambitendency) [7, 9, 13].

Catatonia can be divided into four types [5, 7, 13, 14]. There is the hyperkinetic type of catatonia, characterized by agitation, combativeness [15], verbigeration, stereotypies, echolalia and echopraxia. The stuporous type is characterized by stupor, mutism, posturing and negativism. A third type is lethal or malignant catatonia: an often acutely emerged state involving alterations in consciousness, fever, severe rigidity and autonomous instability. The fourth type is periodic catatonia, characterized by the cyclic return of symptoms of catatonia. These episodes last an average of four to 10 days and can return within weeks or years [9].

The differential diagnosis may be challenging given the current lack of biomarkers and symptom aspecificity. The following conditions need to be considered in the differential diagnosis: malignant antipsychotic syndrome, extrapyramidal side effects of psychopharmacological medication, hyperammonemic encephalopathy [16], acute dystonia, tardive dyskinesia, akathisia, manic episodes, delirium, compulsory movement disorders, conversion disorder, certain compulsions in OCD, serotonin syndrome, locked-in syndrome, vegetative state and stiff person syndrome, kinetic disorders with hypocalcemia, tetanus, strychnine toxicity, rabies and complex partial epilepsy, nonconvulsive status epilepticus [2, 4, 9, 17–19].

In our differential diagnosis we also considered the following: complex partial epileptic seizures [9, 18, 19], postictal akinesia [20], postictal delirium [19], hypoactive delirium [9], nonconvulsive status epilepticus [4, 9], postictal psychosis [21, 22] and non-psychiatric stupor (characterized by immobility, mutism, absence of response to stimuli; this kind of stupor has other causes than catatonia, such as head trauma, anoxia, epilepsy and encephalopathy of unknown origin) [18].

First we would like to address whether it was an ES and not a psychogenic epileptic seizure (PNES) the patient had suffered from. We could not obtain a video EEG, so there is no certainty since video-EEG is the gold standard for such differentiation. We detected however, after the first and second seizures, the last of which was observed by medical staff, a substantial increase in CK which, according to experts, could be a diagnostic instrument for differentiating between ES and PNES [23, 24]. So there is reason to assume it were ES and not PNES.

Another important distinction that must be made in this case is between postictal delirium (occurring in 35% of seizure cases and reported to last sometimes up to 10 days) [25–27] and postictal catatonia as it may substantially influence treatment decision-making [28–30]. Whereas benzodiazepines are used first in cases of catatonia, in delirium such compounds may aggravate symptoms [28–30]. The distinction between the two may be cumbersome given clinical overlap. According to the DSM-5 it is impossible for the two to co-occur since catatonia cannot be diagnosed in the presence of delirium; others question this notion [30]. In addition, Grover [28] found that 12 to 30% of patients with delirium showed symptoms matching the criteria for catatonia.

Another distinction that has to be made is between postictal catatonia and postictal psychosis [21, 22, 31]. Although psychosis as such can be accompanied by catatonic features, this was not the case here as, firstly, except for the psychomotor symptoms, there were no other symptoms of psychosis. Secondly, postictal psychosis is rarely seen with idiopathic generalized epilepsy [21]. And thirdly, postictal psychosis is mostly characterized by delusions of the grandiose or religious type, aggression, pressured speech and excessive emotional response [31].

In our case a diagnosis of catatonia was considered most plausible based on symptomatology (e.g. lack of day-night differences in symptomatology), absence of adverse effects after the lorazepam ‘challenge test’ and repetitive EEGs showing no signs of epileptic activity. Moreover, our observation that the patient remained well-oriented and attentive argued against delirium and that in combination with the absence of delusions or hallucinations none of the above mentioned characteristics of postictal psychosis were present, against postictal psychosis.

The treatment of catatonia initially consists of benzodiazepines [4, 5, 7, 9, 13, 14, 18, 32–35] (effective in 70–80% of all cases) and only when this is ineffective ECT. When ECT is of little help, transcranial magnetic stimulation [18] and NMDA antagonists such as amantadine and memantine [33, 36] may be considered. When these are still ineffective, anticonvulsants can be tried. In some cases, atypical antipsychotics are combined with benzodiazepines [33, 36]. Our patient was treated with valproic acid for epilepsy.

In our case, it was necessary to increase the dose of Lorazepam to 17 mg/d. before we saw clear amelioration of the symptoms. Improvement could in theory also have been attributed to simply time or to the restarted AED since AEDs are also effective in catatonia. But we like to mention that when the AEDs were restarted, the catatonia did not go into remission, and only did so when we augmented the lorazepam to 17 mg/d. Moreover, the fact that the symptoms remained unchanged the first days of occurrence without lorazepam pleads against resolving as a natural course.

The question then arises if ECT would also be a treatment option for our patient. We believe this is since ECT can be given when a patient has a status epilepticus and for catatonia, as mentioned above. There are, to our knowledge, however, no reports of ECT treatment for postictal catatonia.

We searched articles written in Dutch or English in pubmed with the terms ‘post-ictal catatonia’, OR ‘ictal catatonia’ OR ‘catatonia seizures’, OR ‘catatonia postictal state’ OR ‘catatonia and delirium’ as well as cross-references retrieved by our search. We thus found a case series in which four patients developed catatonia during ECT treatment for affective or psychotic disorders [37, 38]. The cause of the catatonia could have been withdrawal of benzodiazepines, besides affective and psychotic disorders, which is supported by the fact that catatonic symptoms disappeared after reinstating benzodiazepines. Two other case reports describe the development of catatonia after ECT, both in patients with a history of bipolar disorder [39, 40], while our patient had no medical history of other psychiatric disorders, except for drug abuse. It is beyond dispute that there is a clear difference between ECT and ES. ECT occurs in a safe environment under controlled circumstances and under narcosis. Furthermore, ECT is often given for conditions that may themselves be accompanied by catatonia. Finally, we found another case report in which catatonia developed postictally, but this patient was known to suffer from schizophrenia [41]. We also found an article by Herman published in 1942 [42], reporting the case of a man stating himself that he suffers from epilepsy. Seizures were, however, not observed and catatonic symptoms were not described clearly.

## Conclusion

To our knowledge, we describe the first case of persistent postictal catatonia, following an observed ES. In this case a daily dose of 17 mg of lorazepam was necessary to attain remission. In patients with bradyphrenia and/or remarkable postictal behaviour we advise to consider not only postictal confusion, but catatonia as well, especially when appearing within 24 h after a seizure. Based on our experience we suggest a gradually increasing dose of lorazepam. This dose of medication should be tapered very gradually as rapid tapering may not only spark catatonia relapse, but a new epileptic seizure too.

Bullets:

1. Catatonia should be considered among those with cognitive and/or movement features that commence postictally.
2. The distinction between delirium and catatonia after an ES may be challenging but has treatment implications.
3. More research into the etiology of catatonia in general and postictal catatonia in particular is highly needed.

## Abbrevations

AED: Anti-epileptic drugs
BFCRS: The Bush Francis catatonia rating scale
CAT Scan: Computer Assisted Tomography
CK: Creatinine kinase
CRP: C-reactive protein
dd: Daily dose
DSM-5: 5th edition of Diagnostic and Statistical Manual of Mental Disorders
ECT: Electroconvulsive therapy
EEG: Electroencephalogram
ES: Epileptic seizures
GABAA: Gamma-aminobutyric acid A
MRI: Magnetic resonance imaging
NMDA: N-methyl D-aspartate
OCD: Obsessive compulsive disorder
PNES: Psychogenic non-epileptic seizures
U/L: Units per liter
